# A Randomized Controlled Trial Examining the Efficacy of Motivational Counseling with Observed Therapy for Antiretroviral Therapy Adherence

**DOI:** 10.1007/s10461-013-0467-3

**Published:** 2013-04-09

**Authors:** Kathy Goggin, Mary M. Gerkovich, Karen B. Williams, Julie W. Banderas, Delwyn Catley, Jannette Berkley-Patton, Glenn J. Wagner, James Stanford, Sally Neville, Vinutha K. Kumar, David M. Bamberger, Lisa A. Clough

**Affiliations:** 1 HIV Research Group, Department of Psychology, University of Missouri—Kansas City, 5030 Cherry Street, Ste 310, Kansas City, MO 64110 USA; 2 Department of Biomedical and Health Informatics, School of Medicine, University of Missouri—Kansas City, Kansas City, MO USA; 3 School of Medicine, University of Missouri—Kansas City, Kansas City, MO USA; 10 Smoking and Motivation Research Lab, Department of Psychology, University of Missouri—Kansas City, Kansas City, MO USA; 11 Community Health Research Group, Department of Psychology, University of Missouri—Kansas City, Kansas City, MO USA; 4 RAND Corporation, Santa Monica, CA USA; 5 Section of Infectious Diseases, Department of Medicine, University of Missouri—Kansas City, Kansas City, MO USA; 6 Kansas City CARE Clinic, Kansas City, MO USA; 7 Division of Infectious Diseases, Kansas City Veterans Affairs Medical Center, Kansas City, MO USA; 8 Division of Infectious Diseases, University of Kansas Medical Center, Kansas City, KS USA

**Keywords:** Adherence, HIV/AIDS, ART, Motivational Interviewing, Directly Observed Therapy

## Abstract

This study determined whether motivational interviewing-based cognitive behavioral therapy (MI-CBT) adherence counseling combined with modified directly observed therapy (MI-CBT/mDOT) is more effective than MI-CBT counseling alone or standard care (SC) in increasing adherence over time. A three-armed randomized controlled 48-week trial with continuous electronic drug monitored adherence was conducted by randomly assigning 204 HIV-positive participants to either 10 sessions of MI-CBT counseling with mDOT for 24 weeks, 10 sessions of MI-CBT counseling alone, or SC. Poisson mixed effects regression models revealed significant interaction effects of intervention over time on non-adherence defined as percent of doses not-taken (IRR = 1.011, CI = 1.000–1.018) and percent of doses not-taken on time (IRR = 1.006, CI = 1.001–1.011) in the 30 days preceding each assessment. There were no significant differences between groups, but trends were observed for the MI-CBT/mDOT group to have greater 12 week on-time and worse 48 week adherence than the SC group. Findings of modest to null impact on adherence despite intensive interventions highlights the need for more effective interventions to maintain high adherence over time.

## Resumen

Esta investigación determina si la Terapia Cognitivo-Conductal basada en la Entrevista Motivacional (MI-CBT) combinada con Terapia de Observación Directa modificada (MI-CBT/mDOT) es más efectiva que una intervención que solo incluye MI-CBT o el cuidado estándar (SC) para aumentar la adherencia a medicamento a largo plazo. Llevamos a cabo un periodo de prueba controlado de tres ramas aleatorias por 48 semanas, el cual incluye un seguimiento electrónico continuo de adherencia a medicamento. Un total de 204 participantes VIH-positivo fueron aleatoriamente asignados a 10 sesiones de terapia MI-CBT con mDOT por 24 semanas, 10 sesiones de terapia MI-CBT sola, o SC. Modelos de regresión Poisson de efectos mixtos revelaron efectos significativos de interacción de la intervención por tiempo en no-adherencia definida por el porciento de dosis no-tomadas (IRR = 1.011, CI = 1.000–1.018) y el porciento de dosis no-tomadas a tiempo (IRR = 1.006, CI = 1.001–1.011) a 30 días antes de cada evaluación. No hubo diferencias significantes entre grupos, pero el grupo de MI-CBT/mDOT tubo tendencia a tener mejor adherencia a tomar el medicamento a tiempo a las 12 semanas y peor adherencia a las 48 semanas comparado con el grupo SC. Estos resultados de efectos nulo-a-modesto en la adherencia a pesar de intervenciones intensivas, recalcan la necesidad de tener más intervenciones efectivas que ayuden a mantener una alta adherencia a largo plazo.

## Introduction

Widespread use of antiretroviral therapy (ART) has resulted in significant numbers of patients achieving durable viral load (VL) suppression and reduced morbidity and mortality [1, 2]. The benefits of ART are clear [3], however its initial success and long-term effectiveness are dependent on strict regimen adherence, which is difficult and often not sustainable over time [4, 5].

Most studies to promote ART adherence have tested cognitive-behavioral techniques for increasing knowledge and skills for adherence with several demonstrating promise [6–8]. Some of the most effective trials have combined motivational interviewing (MI) techniques with cognitive-behavioral treatment (CBT) techniques to produce comprehensive ART adherence counseling interventions [7, 9–12]. Others which have focused on providing external supports for adherence, like modified directly observed therapy (mDOT), have also shown some promise [13–16] although null results and high burden have also been noted [17]. Both approaches are potentially cost effective [18] and adaptable to a variety of patients and settings [19, 20], however no studies have assessed the combined effect of motivational interviewing-based cognitive behavioral therapy (MI-CBT) counseling and mDOT approaches. Evidence of whether such a combination has an additive effect on adherence and clinical outcomes would inform the allocation of limited resources for adherence enhancement in community practice.

This paper reports findings from a three arm, randomized controlled trial that examined whether MI-CBT counseling combined with mDOT (MI-CBT/mDOT) is more effective than counseling alone (MI-CBT) or standard care (SC) for increasing adherence to ART over 48 weeks among HIV-positive community clinic patients. The secondary aim was to evaluate intervention effects on suppression of VL.

## Methods

### Procedures

Data were collected from December 2004 to August 2009 at six outpatient clinics in Kansas City. Eligible participants were HIV-positive and were either: starting ART for the first time; making a change to their regimen; or having self-reported adherence problems (confirmed by provider documentation and/or HIV RNA >1,000 copies/mL). Participants were also >18 years of age and English speaking. Participants were excluded if they were pregnant, had an acute illness that would interfere with their ability to participate, did not self-administer their ART, or did not live within a 70-mile radius of the project office. Approval for the study was obtained from the appropriate Institutional Review Boards.

Participants completed the baseline assessment that included demographic, adherence, psychosocial and medical variables via Audio Computer Assisted Self Interview and were randomized on a 1:1:1 ratio. Participants Group random assignment was stratified by ART naïve/experienced and by clinic. An adapted version of the Alcohol and Substance Use Inventory [21] was used to collect data on the frequency and quantity of substance use. Participants reported their frequency of binge drinking over the past 30 days and their use of drugs (i.e., illicit, prescription or over the counter drugs taken in excess of the directions) from seven specific drug classifications (e.g., marijuana, cocaine, opiates, and amphetamines) over the past 3 months. Binge drinking was defined as having six or more alcoholic drinks during a single drinking occasion. The Center for Epidemiologic Studies Depression Scale (CES-D; [22]) was used to assess depressive symptoms. Intervention and data collection activities were completed by different project staff.

Each participant was given an electronic drug monitor (EDM; http://www.aardex.ch) to be used continuously throughout the study to track adherence. When participants were on more than one ART medication, we monitored adherence to the drug with the most complex dosing schedule or to the drug that was expected to have the most severe side effects if dosing schedules were identical for all medications. Participants continued to receive routine medical care and were scheduled for monthly EDM downloads and follow-up assessments at 12, 24, 36, 48 weeks. Participants were provided up to $165 for completion of assessment visits (i.e., $20 at the baseline, 12 and 36 week visits; $40 at the 24 week visit; and $65 at the 48 week visit). To increase identification with the study and retention, participants were also offered attractive Project MOTIV8 logo items (e.g., t-shirts and water bottles) and a study completion certificate.

### Standard Care (SC)

Participants assigned to SC received medical care and adherence counseling as usual from their clinic providers. A multi-modal assessment (i.e., randomly selected convenience subsample of medical record abstractions, provider surveys, and patient surveys) was employed to evaluate the SC delivered by the clinics where the majority (82 %) of participants received care. We assessed SC recommended monitoring of patients receiving ART including clinic visits and laboratory tests at anticipated intervals to assess effectiveness and safety, side effect monitoring and management, and continual adherence assessment and counseling [23–25]. Data was collected prior to initiation of participant recruitment and repeated when the final participants were completing the study. No differences between clinics were noted, however clinic care changed in accordance with updated recommended guidelines at approximately the same time at all sites [26].

### Motivational Interviewing Based Cognitive Behavioral Counseling (MI-CBT)

Participants assigned to the MI-CBT and MI-CBT/mDOT arms received care as usual from their clinic providers and met with project staff for six face-to-face MI-CBT counseling sessions (weeks 0, 1, 2, 6, 11 and 23) and four telephone sessions (weeks 4, 9, 15, and 19).

Our MI-CBT intervention included the use of MI; [27] techniques to increase motivation and confidence for change as well as the use of cognitive-behavioral approaches delivered in an MI-consistent style to enhance knowledge and build skills (e.g., self-monitoring, problem-solving, talking to your doctor) for adherence [28]. On average, sessions lasted 25 min. Counselors were Master’s degree level professionals trained and supervised by a licensed clinical psychologist. Counselors digitally recorded sessions and received ongoing weekly supervision in which randomly selected session tapes were coded for fidelity using a 26-item coding scheme adapted from our prior work [29]. Counselors maintained high fidelity throughout the study with an average rating of 6.2 (SD = 1) on an overall summary item (“Overall, how well did the counselor conduct this session?”) scored on a 7-point scale ranging for poor [1] to excellent [7].

### Modified Directly Observed Therapy (mDOT)

Participants in the MI-CBT/mDOT intervention arm received care as usual from their clinic providers, the MI-CBT counseling described above, and daily mDOT visits (Monday through Friday) from baseline through week 16. Visits were tapered at week 17 until they ceased at week 24. Each mDOT visit (average length 5.2 min) was conducted at a location and planned dose time that was most convenient for the participant.

As with participants in the other groups, MI-CBT/mDOT participants obtained their own ART medications and kept one medication in the EDM bottle. Their remaining ART medications were transferred to study staff. MI-CBT/mDOT participants were always in possession of a 1-week emergency back-up supply of all of their medications for use if a visit was missed. At each visit, ingestion of an ART dose was observed, remaining daily doses were delivered (weekend doses were delivered on Fridays), and participants reported on their adherence to all unobserved ART doses since the last mDOT visit using personal digital assistants (PDA’s). Initially all of these visits were conducted in person, however due to medication regimen changes (i.e., more once per day ART), late night dosing (e.g., efavirenz), and the inclusion of participants who lived outside of the catchment area, the mDOT protocol was revised over the course of the study to include in-person as well as ‘phone contacts’ (participant ingested medication during a study staff initiated phone call at the predetermined dose time), ‘med delivery’ (meds delivered outside of target dosing time and participant reported by phone/text when ingested), and ‘PDA visits’ (meds delivered outside of target dosing time and participant retrospectively reported on all unobserved doses using PDA). These adaptations made our version of mDOT distinct from others tested in previous studies. During weeks 22 and 23 staff ensured that participants could accurately fill their pill boxes and then returned all remaining medications to participants at the last mDOT visit.

### Outcomes Measures

Raw EDM adherence data were cleaned to ensure that no patient had greater than 100 % adherence in any 24 h period. Periods where participants were unable to use the EDM cap due to hospitalization, physician ordered medication holiday, or incarceration were also excluded. Each opening was then evaluated to determine whether or not the dose was ‘on time.’ Cap openings for participants on once daily and twice a day dosing schedules were on time if the opening occurred within ±2 h of the scheduled dosing time. Summary adherence variables of percent of doses taken and percent of doses taken on time for the 30 day period before each assessment visit were then calculated for each participant.

Study staff abstracted HIV VL from medical records if they were available, generally within ±30 days of assessment visits, otherwise blood draws were conducted at the assessment visits. In order to accommodate VL testing standards over the entire study period, data were dichotomized as undetectable (<400 copies/mL) or detectable (>400 copies/mL).

### Dose of Intervention

To explore dose response effects, a variable was constructed that reflected how much intervention each participant received. Participants in the SC arm were given a “0” as they received no intervention. MI-CBT and mDOT dose was computed as the percent of possible sessions that were completed. The final intervention dose variable reflected the MI-CBT dose for those assigned to the MI-CBT arm and the sum of the MI-CBT and mDOT doses for those assigned to the MI-CBT/mDOT arm.

### Statistical Methods

Primary and secondary outcomes were analyzed using an intention-to-treat analysis. Both percent taken and percent on time adherence outcomes violated Mauchly’s test of sphericity and evidence highly negatively skewed distributions making our planned least squares regression approach problematic. Based on recommendations of Gardner [30] for managing rate data, Poisson mixed effects regression models were fit to assess effects of intervention group over time (intervention group and observation period as fixed effects) clustered within individual (participants as a random effect) on rate of adherence. To better meet the assumptions of the Poisson mixed model, data were reverse coded by subtracting percent taken adherence rate from 100 to create a percent of doses not-taken outcome. The same was done to produce a percent of doses not-taken on time outcome. The Poisson mixed effects model provides an appropriate mechanism for handling repeated measures rate data while allowing for the inclusion of available data from participants who had missing evaluation time points. This was an additional advantage as we had partial missing data from 42 participants. Analyses were conducted using STATA 11.0 SE (StataCorp LP, College Station TX) to derive full maximum-likelihood and variance estimates with model assumptions confirmed through the analysis of residuals. For all models, the likelihood ratio test was used for comparison of nested models (random intercept and random coefficients models). The regression values are reported as incidence rate ratios (IRR; predicted values that are natural logarithms of relative risk) modeling non-adherence.

Additionally, models were fit to explore the effect of dose of intervention on non-adherence. Participants assigned to the SC group were not included in the dose analyses. In order to explore the effect of increasing dose of intervention, dose was categorized into quintiles (from lowest to highest) for analyses. As with the other outcomes, mixed effects Poisson models were fit.

A logistic mixed effect regression model was fit to evaluate suppression of VL to undetectable as a function of intervention group while controlling for observation period. Odds ratios (OR) are reported for undetectable VL (binary data) using robust standard errors to provide conservative estimates of statistical significance.

## Results

### Recruitment and Attrition

**Fig. 1:**
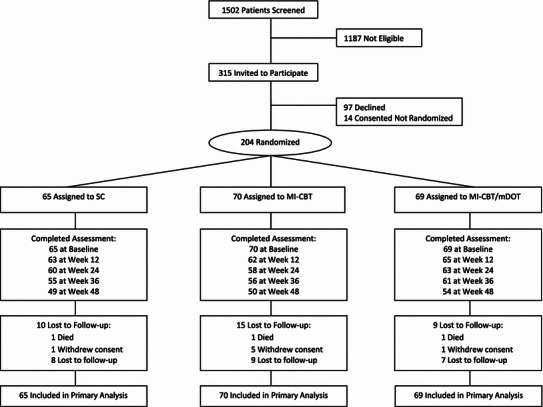
Study flowchart

Of the 1,502 patients screened for eligibility, 1,187 (79 %) did not meet study eligibility criteria, because they were not switching medications, not on medications, not experiencing adherence problems, were pregnant, or had an opportunistic infection (see Fig. 1). Of the 315 who met eligibility criteria, 97 (31 %) were approached but declined to participate. The primary reasons for refusal were lack of interest in research, being too busy, and anticipating an out of town move.

Attrition over the 48 week study was 16.7 % and did not differ by study arm. A total of 901 (88 % of total possible) assessment sessions were completed. Data were collected from all 204 participants at baseline, 181 at 12 and 24 weeks, 165 at 36 weeks, and 170 participants at week 48. Sociodemographic characteristics of participants with complete self-report data (75 %), those who missed a single assessment (9 %), and those who missed two or more assessments (16 %) did not differ (all *p*s > .10).

### Participant Characteristics and Evaluation of Randomization

Participants ranged in age from 18 to 65, 47 % were heterosexual, and 91 % had stable housing (see Table 1). At baseline, participants had been diagnosed with HIV for an average of 8 years, and most were on once (*n* = 108, 53 %) or twice (*n* = 89, 44 %) daily ART regimens with a small percentage (*n* = 7, 3 %) on a thrice per day regimen. There were no baseline differences between groups on any sociodemographic, regimen, or predictor variables (see Table 1; all *p*s > .10).

**Table 1:** Demographic characteristics of the sample at baseline

Variables			Treatment			
	SC *n* = 65		MI-CBT *n* = 70		MI-CBT/mDOT *n* = 69	
	*n*	% or mean (SD)	*n*	% or mean (SD)	*n*	% or mean (SD)
Age—Mean (SD)	65	40.4 (8.2)	70	40.8 (9.6)	69	39.9 (10.7)
Male gender at birth (%)	50	76.9 %	50	71.4 %	55	79.7 %
Ethnicity/race Hispanic (%)	4	6.2 %	8	11.4 %	7	10.1 %
African American (%)	38	58.5 %	35	50.0 %	43	62.3 %
White (%)	21	32.3 %	22	31.4 %	22	31.9 %
Other (%)	6	9.2 %	13	18.6 %	4	5.8 %
Income < $12,000/year (%)	40	69.0 %	44	67.7 %	41	66.1 %
Education						
Less than high school degree (%)	17	26.2 %	14	20.0 %	15	21.7 %
High school graduate/GED (%)	21	32.3 %	20	28.6 %	21	30.4 %
More than high school degree (%)	27	41.5 %	36	51.4 %	33	47.8 %
Work status						
Working full time/part time (%)^a^	17	26.2 %	23	32.9 %	20	29.0 %
On disability (%)	31	47.7 %	25	35.7 %	21	30.4 %
No income (%)	9	13.9 %	14	20.0 %	21	30.4 %
Married/committed relationship	15	23.4 %	15	21.7 %	20	29.0 %
Covered by private insurance (%)	5	7.7 %	5	7.1 %	8	11.6 %
CD4—% below 200 cells	31	47.6 %	25	36.2 %	33	47.8 %
ART Naïve	21	32.3 %	24	34.3 %	24	34.8 %
Illicit drug use in last 3 months (%)	29	44.6 %	29	42.0 %	30	43.5 %
Binge drinking in last 30 days (%)	15	23.1 %	11	15.9 %	14	20.3 %
CES-D total score >16 (%)	35	53.8 %	39	56.5 %	33	47.8 %

^a^Categories are not mutually exclusive and three participants both worked and collected disability. Results for some baseline data for the EC group is based on 69/70 participants as a portion of one participant’s baseline evaluation was lost during data transfer

### Uptake of the Interventions

A total of 1,170 (77 % of the total possible) MI-CBT counseling sessions were completed by participants in the MI-CBT and MI-CBT/mDOT study arms. The average number of counseling sessions was similar between participants in the MI-CBT (8.40, SD = 2.7) and MI-CBT/mDOT (8.43, SD = 2.9) arms (*p* = .94).

A total of 4,139 (61 % of total possible) mDOT visits were completed. Of the total, 1,927 mDOT visits were completed in person, 1,324 were completed via phone, 764 visits were med delivery, and 124 were PDA visits. The average rate of completion in the MI-CBT/mDOT arm was 60 (SD = 28) of the 98 total possible mDOT visits. A Poisson mixed effects regression model revealed that average non-adherence was not significantly different for participants who received the majority of their mDOT sessions in person or via phone (IRR = 1.46, SE = .85, 95 % CI = .47–4.57, *p* = .51). Average dose of intervention was 76.4 (SD = 24.0) for participants in the MI-CBT arm and 148.7 (SD = 52.7) for MI-CBT/mDOT participants.

### Missing Data

Because EDM data is recorded continuously and stored, prior adherence data can be collected at any point that the cap is available. For this reason we were sometimes able to capture EDM adherence data that coincided with a missed assessment visit. For week 12, we had 91 % of all possible EDM data, 89 % at week 24, 86 % at week 36, and 81 % at week 48. Comparison of participants with complete versus incomplete EDM data identified no significant differences in sociodemographic characteristics. In total, 24 % of VL data across all assessment visits were missing. Specifically, we had no missing VL data at baseline, 22 % missing at 12 weeks, 23 % missing at 24 weeks, 41 % missing at 36 weeks, and 34 % missing at 48 weeks.

### Evaluation of Intervention Effects

Table 2 summarizes mean adherence for both percent of doses taken and percent of doses taken on time at each assessment point and by group. Analyses were based on the intent-to-treat sample of 204 participants. Of these, 14 subjects had insufficient data over time to be included in the analyses exploring intervention effects (i.e., data for fewer than three of the four time points). Four Poisson mixed regression models were fit to assess non-adherence. Models were fit to test the effect of intervention group and observation period on non-adherence defined as percent of doses not-taken (Model 1) and percent of doses not-taken on time (Model 2) in the 30 days preceding each assessment visit (Table 3). Results of Model 1 revealed a statistically significant, albeit small, interaction effect of intervention over time (IRR 1.011, 95 % CI = 1.004–1.018, *p* = .003) on non-adherence indicating that the change in adherence over time differed between the study groups. Model 2 had similar findings to Model 1 with a statistically significant, yet small, interaction effect between intervention group and observation period (IRR = 1.006, 95 % CI = 1.001–1.011, *p* = .015). In both models, neither the intervention group nor observation period main effects were significant predictors of non-adherence; however, a trend emerged for the main effect of intervention group on percent of doses not-taken on time (IRR .782, 95 % CI = .594–1.029, *p* = .079). Inspection of the means in Table 2 revealed a cross-over in adherence trends over time for both percent of doses and percent of doses on time such that the MI-CBT/mDOT intervention group started with the highest adherence through 24 weeks and then declined to end up with the lowest adherence at 48 weeks. The MI-CBT intervention group was slightly higher than SC at 12 weeks, but also declined over time to below SC. SC remained relatively consistent throughout the study showing the least amount of decline in adherence over time. As evidenced by the large standard deviations, adherence varied greatly within groups.

**Table 2:** Percent adherence and clinical outcomes data by intervention arm

EDM % doses taken	All participants *N* = 204	SC *n* = 65	MI-CBT *n* = 70	MI-CBT/mDOT *n* = 69
	M (SD)	M (SD)	M (SD)	M (SD)
12 weeks	78.5 (29.8)	75.3 (32.6)	77.6 (32.1)	82.5 (23.6)
24 weeks	73.0 (32.2)	73.5 (32.5)	70.9 (34.5)	74.4 (30.1)
36 weeks	70.3 (32.4)	74.7 (30.3)	69.3 (33.1)	67.2 (33.7)
48 weeks	69.6 (32.4)	75.5 (29.7)	69.3 (32.6)	64.6 (34.4)

EDM % doses taken on time				
12 weeks	67.7 (31.7)	62.3 (33.6)	67.7 (32.7)	73.1 (28.2)
24 weeks	61.4 (33.1)	58.6 (33.1)	61.1 (34.4)	64.2 (32.0)
36 weeks	56.3 (33.5)	57.6 (33.2)	56.5 (34.0)	54.9 (33.8)
48 weeks	55.5 (33.4)	58.1 (32.5)	56.9 (34.1)	52.0 (34.1)

% Participants HIV-1 RNA VL <400 copies/mL	% (*n*)	% (*n*)	% (*n*)	% (*n*)
Baseline	6.4 (204)	4.6 (65)	8.6 (70)	5.8 (69)
12 weeks	70.4 (159)	72.0 (50)	78.2 (55)	61.1 (54)
24 weeks	73.2 (157)	71.4 (49)	70.6 (51)	77.2 (57)
36 weeks	70.2 (121)	71.1 (38)	72.5 (40)	67.4 (43)
48 weeks	73.3 (135)	73.8 (42)	80.0 (45)	66.7 (48)

*EDM* electronic drug monitored

**Table 3:** Poisson random coefficients model: incident rate ratios for intervention group, observation period, and the group by time interaction for the level of non-adherence

Variables: models 1 and 2	Percent not taken^a^			Percent not taken on time^a^		
	IRR	95 % CI	*p*	IRR	95 % CI	*p*
Observation period (12 weeks reference)	.990	(.974, 1.005)	.195	1.000	(.989, 1.011)	.967
Intervention group (SC reference)	.797	(.541, 1.175)	.252	.782	(.594, 1.029)	.079
Interaction	1.011	(1.004, 1.018)	.003	1.006	(1.001, 1.011)	.015

^a^ *N* for full models: 709 observations on 190 participants

Post hoc ANOVA analyses comparing group means at each individual assessment point revealed no significant group differences, but did reveal trends for the MI-CBT/mDOT group to have greater on-time adherence at 12 weeks [F (1, 119) = 3.67, *p* = .058] and worse dose adherence at 48 weeks [F (1, 110) = 3.21, *p* = .076] than the SC group.

### Dose–Response Relationship

Two additional models were fit to examine the dose–response effect of increasing intervention dose (by lowest to highest quintiles) on non-adherence for subjects in the MI-CBT and MI-CBT/mDOT groups while controlling for observation period and the interaction of observation period and dose. For both outcomes, only the results of the main effects analyses are reported as both interactions were not significant and their inclusion did not significantly improve model fit. Results for both percent not-taken (Model 3) and percent not-taken on time (Model 4) showed that while non-adherence increased over time (IRR’s greater than 1.0), having more exposure to the intervention was associated with less non-adherence (IRR’s less than 1.0) (Table 4). Moreover, there was a gradient effect such that each increasing quintile of dose was predictive of a comparable reduction in non-adherence.

**Table 4:** Poisson random coefficients model: incident rate ratios for intervention dose and observation period for the level of non-adherence for participants in the intervention groups

Variables: models 3 and 4	Percent not taken^a^			Percent not taken on time^a^		
	IRR	95 % CI	*p*	IRR	95 % CI	*p*
Observation period (12 weeks reference)	1.016	(1.009, 1.023)	<.001	1.015	(1.010, 1.020)	<.001
Intervention dose (first quintile reference)						
Second quintile	.318	(.110, .923)	.035	.473	(.217, 1.035)	.061
Third quintile	.161	(.052, .494)	.001	.359	(.158, .816)	.014
Fourth quintile	.044	(.015, .129)	<.001	.129	(.058, .284)	<.001
Fifth quintile	.034	(.011, .100)	<.001	.077	(.035, .171)	<.001

^a^ *N* for full models: 482 observations on 129 participants

### Analyses of Viral Load

Table 2 displays the percentage of participants with an undetectable VL at each assessment point by group. Across all groups the percentage of individuals with an undetectable VL increased from baseline to 12 weeks and then remained relatively constant through 48 weeks. A logistic mixed effects regression model was fit to examine the effects of intervention group and observation period on VL (undetectable vs. detectable) over time. No significant relationship between intervention group and the likelihood of having an undetectable VL was observed (OR = .94, 95 % CI = .67–1.32, *p* = .72). Irrespective of group, the odds of being undetectable increased significantly over time (OR = 1.08, 95 % CI = 1.07–1.10, *p* = .0001).

## Discussion

The primary aim of the study was to explore the efficacy of MI-CBT counseling combined with mDOT as compared to MI-CBT counseling alone or SC for increasing adherence to ART. The primary analyses revealed a significant interaction effect indicating that adherence patterns over time differed between the groups. The pattern of results suggest that the MI-CBT/mDOT intervention may have had its greatest impact at 12 weeks coinciding with the most intensive portion of the intervention, and then declined more steeply than SC and MI-CBT as treatment was tapered and withdrawn. This is consistent with other studies of behavioral counseling that observe a decline at the conclusion of active treatment [6, 8, 16, 31]. Given the modest magnitude of the interaction effect and the lack of significant differences between groups at any time point, it is also possible that neither of the intervention arms had any true impact on adherence and the observed fluctuation is merely a function of being part of a study (i.e., Hawthorne Effect) followed by regression to the mean over time. It is also possible that adaptations made to the mDOT protocol could have diminished the impact of the mDOT portion of the MI-CBT/mDOT intervention. Another possibility is that after initially supporting increased adherence, withdrawing this intensive intervention actually had an iatrogenic effect and drove down adherence. We could identify no literature support for this type of rebound effect in MI-CBT styled interventions, however, there is some evidence of steep declines in adherence after removal of mDOT interventions [32].

Significant dose response relationships were observed indicating that participants who received more dose of the interventions had better adherence. These findings increase our confidence that the intervention may have had an impact on some participants’ ability to adhere. While this finding could be attributable simply to the five mDOT visits per week where ingestion of doses were directly observed, nearly half of participants were on twice or thrice a day dosing schedules meaning that they self-administered more than half of their doses outside of mDOT visits. These findings are more consistent with the notion that more intervention is associated with better adherence [11, 33–37]. Nevertheless, the lack of significant main effect findings underscores how even intensive interventions can fail to substantially improve adherence over high quality SC.

Future research should focus on adapting established efficacious behavioral interventions [6, 31] for use in real world settings. The results of this study suggest that these adjunctive interventions will likely need to be intensive and long in duration to have positive impacts on health outcomes. Use of technology (e.g., text messaging via cell phones) to provide the high levels of contact provided in established effective behavioral interventions at low cost has shown some promise (e.g., [38]) and will be essential for effective dissemination. Linking interventions to real-time drops in adherence assessed remotely using EDM devises [39] may be particularly useful and allow for matching of intervention strength and style to patient needs and preferences which has shown promise in the treatment of substance abuse [40]. Research of this kind will also assist in the identification of patients in need of the most intensive interventions while providing easily accessible data for establishing the cost of sustained intervention (e.g., [41]).

We found no evidence of a relationship between intervention group and the likelihood of having an undetectable VL. The lack of a direct effect is consistent with recent meta-analytic findings [16, 17]. In our study the percentage of participants with undetectable VL rose quickly and then remained relatively stable across all arms. The high rate of undetectable VL in the SC arm despite consistently low levels of adherence are consistent with research demonstrating that modern ART is more forgiving of lower levels of adherence [42], but likely diminished our ability to detect any intervention effect.

Study limitations include our inclusion of individuals without documented adherence problems which likely reduced our ability to demonstrate the full amplitude of intervention effects [43] and lack of baseline adherence data. Analyses were also hampered by missing VL data and our failure to exclude participants who had already had undetectable VL.

In spite of these limitations, this rigorous study contributes to our understanding of the impact of intensive ART adherence interventions. Although intensive, the interventions tested here produced a modest to null impact on adherence. The findings highlight the need for more effective interventions to maintain high adherence over time. The findings of an initial uptick followed by steep decline in adherence in the MI-CBT/mDOT intervention arm also suggest some caution maybe warranted in using mDOT based interventions.
